# Surfactant Attenuates Air Embolism-Induced Lung Injury by Suppressing NKCC1 Expression and NF-κB Activation

**DOI:** 10.1007/s10753-020-01266-1

**Published:** 2020-10-22

**Authors:** Chou-Chin Lan, Yao-Kuang Wu, Chung-Kan Peng, Kun-Lun Huang, Chin-Pyng Wu

**Affiliations:** 1 Division of Pulmonary Medicine, Taipei Tzu Chi Hospital, Buddhist Tzu Chi Medical Foundation, New Taipei City, Taiwan; 2 School of Medicine, Tzu-Chi University, Hualien, Taiwan; 3 Division of Pulmonary Medicine, Tri-Service General Hospital, Taipei, Taiwan; 4 Institute of Undersea and Hyperbaric Medicine, National Defense Medical Center, Taipei, Taiwan; 5 Department of Critical Care Medicine, Landseed International Hospital, Tao-Yuan, Taiwan

**Keywords:** air embolism, Na-K-Cl cotransporter isoform 1, lung injury, surfactant

## Abstract

Excessive amounts of air can enter the lungs and cause air embolism (AE)-induced acute lung injury (ALI). Pulmonary AE can occur during diving, aviation, and iatrogenic invasive procedures. AE-induced lung injury presents with severe hypoxia, pulmonary hypertension, microvascular hyper-permeability, and severe inflammatory responses. Pulmonary AE-induced ALI is a serious complication resulting in significant morbidity and mortality. Surfactant is abundant in the lungs and its function is to lower surface tension. Earlier studies have explored the beneficial effects of surfactant in ALI; however, none have investigated the role of surfactant in pulmonary AE-induced ALI. Therefore, we conducted this study to determine the effects of surfactant in pulmonary AE-induced ALI. Isolated-perfused rat lungs were used as a model of pulmonary AE. The animals were divided into four groups (*n* = 6 per group): sham, air embolism (AE), AE + surfactant (0.5 mg/kg), and AE+ surfactant (1 mg/kg). Surfactant pretreatment was administered before the induction of pulmonary AE. Pulmonary AE was induced by the infusion of 0.7 cc air through a pulmonary artery catheter. After induction of air, pulmonary AE was presented with pulmonary edema, pulmonary microvascular hyper-permeability, and lung inflammation with neutrophilic sequestration. Activation of NF-κB was observed, along with increased expression of pro-inflammatory cytokines, and Na-K-Cl cotransporter isoform 1 (NKCC1). Surfactant suppressed the activation of NF-κB and decreased the expression of pro-inflammatory cytokines and NKCC1, thereby attenuating AE-induced lung injury. Therefore, AE-induced ALI presented with pulmonary edema, microvascular hyper-permeability, and lung inflammation. Surfactant suppressed the expressions of NF-κB, pro-inflammatory cytokines, and NKCC1, thereby attenuating AE-induced lung injury.

## INTRODUCTION

Pulmonary air embolism (AE) occurs when air enters the circulatory system and lungs [4]. There are a lot of iatrogenic invasive procedures that can lead to pulmonary AE, including radical neck dissection, laparoscopic surgery, therapeutic bronchoscopy, central venous access, and hemodialysis catheters*.* [4, 7]. Pulmonary AE is also known to occur in pilots during high-altitude flights and divers undergoing decompression after hyperbaric exposure.

Under normal conditions, small amounts of air are usually absorbed from the pulmonary vasculature. However, when the amounts of air exceed the absorption capability of the lung, acute lung injury (ALI) can result and is termed AE-induced ALI [19]. The AE-induced ALI presents with pulmonary edema, pulmonary hypertension, impaired gas exchange, and profound hypoxia [7]. AE-induced ALI is therefore a serious complication resulting in significant morbidity and mortality [4, 7].

The mechanisms of AE-induced ALI are multiple factors [12, 14, 22]. Pulmonary AE causes physical obstruction of microvasculature, leads to permeability changes, and releases inflammatory mediators. Air bubbles irritate the pulmonary endothelium and induce a series of inflammatory responses [14]. Neutrophils also play an important role in the pathogenesis of AE-induced ALI [22] by interacting with the air emboli and activating inflammatory responses, causing the release of reactive oxygen species, proteases, and cytokines and up-regulate the production of adhesion molecules and result in further lung damage [12]. Together, these factors lead to lung injury with vascular hyper-permeability, pulmonary edema, and poor gas exchanges [12, 14, 22].

Surfactant is abundant in the lungs. It alters respiratory mechanics by its surface-active properties, thereby lowering surface tension. Surfactant dysfunction contributes to the disturbed physiology in ALI with progressive loss of aerated volume and exacerbation of ventilation/perfusion mismatch [6]. Both animal and human studies suggested that surfactant therapy is effective for ALI and acute respiratory distress syndrome (ARDS) [2, 15, 25]. Amigoni et al. showed that surfactant is effective in children and infants with ARDS [2]. Nakajima et al. suggested that surfactant replacement reduced the levels of inflammatory cytokines and secretory phospholipase A_2_ in pigs with gastric acid aspiration-induced lung injury [15]. While exogenous surfactant administration seems to improve lung function in ALI [25], the effects of surfactant in AE-induced ALI are still unclear.

Alveolar fluid regulation plays a critical role in the development of pulmonary edema in lung injury. The pulmonary ion channel Na-K-Cl cotransporter isoform 1 (NKCC1) is important in alveolar fluid regulation. NKCC1 locates basolaterally in the lung epithelium that mediates a net influx of ions into alveolar cells [1]. The chemical gradient that creates the driving force for water transport is therefore mediated primarily by NKCC1 [1]. Besides, NKCC1 is known to regulate lung inflammation [16, 24]. One previous study showed that mice lacking NKCC1 are protected from bacterial pneumonia [16]. Previously, we also suggested that NKCC1 plays an important role in IR-induced lung injury, in that increased expression of NKCC1 leads to more severe ALI. In the current study, we further investigated the role of NKCC1 in AE-induced lung injury.

Surfactant and NKCC1 are both important in the regulation of lung water and inflammation. We hypothesized that the administration of surfactant should be a benefit for AE-induced lung injury. However, studies exploring surfactant and NKCC1 in AE-induced lung injury are lacking. Therefore, this study aimed to determine the effects of surfactant and changes in NKCC1 in AE-induced ALI.

## MATERIALS AND METHODS

### Isolation and Perfusion of the Lungs

The National Science Council and Animal Review Committee of the National Defense Medical Center (Taipei, Taiwan) approved the protocol for this study. The animals were cared for in accordance with the “Guide for the Care and Use of Laboratory Animals” published by the US National Institutes of Health.

Procedures regarding the preparation of isolated-perfused lungs *in situ* in the chest were as previously described [11]. Male Sprague-Dawley rats were anesthetized through intraperitoneal injection of pentobarbital sodium (50 mg/kg). After confirmation of deep anesthesia, tracheostomy was performed and a cannula was inserted into the trachea. The lungs were ventilated with a humidified gas mixture containing 5% CO_2_ in air at a frequency of 60 cycle/min, tidal volume of 3 mL, and end-expiratory pressure of 1 cm H_2_O. Median sternotomy was performed and heparin (1 U/g of body weight) was injected into the right ventricle.

A peristaltic pump (Model 1203, Harvard Apparatus) was used to perfuse the lungs with re-circulated perfusate comprising blood mixed with physiological salt solution (119 mM NaCl, 4.7 mM KCl, 1.17 mM MgSO_4_, 22.6 mM NaHCO_3_, 1.18 mM KH_2_PO_4_, 1.6 mM CaCl_2_, 5.5 mM glucose, and 50 mM sucrose). Bovine albumin (4 g/dL) was added to maintain the osmolarity of the perfusate. A cannula was placed in the left atrium *via* the left ventricle to collect the effluent perfusate for re-circulation.

The perfusion rate and temperature were maintained at 8–10 mL/min (by a roller pump) and 37 °C (by a water bath), respectively. The preparation was placed on an electronic balance with the isolated lungs remaining *in situ*.

### Experimental Protocols and Induction of Air Embolism-Induced Acute Lung Injury

The animals were divided into four groups (*n* = 6 per group): sham, AE, AE + surfactant (0.5 mg/kg), and AE + surfactant (1 mg/kg). Pretreatment surfactant was administered before induction of AE. Pulmonary AE was induced through infusion of 0.7 cc air through the pulmonary artery catheter.

All groups were studied for pulmonary microvascular permeability (Kf), alveolar fluid clearance (AFC), lung histopathology, lung weight/body weight ratio (LW/BW), lung wet/dry weight ratio (W/D), and levels of tumor necrosis factor-alpha (TNF-α), chemokine (C-X-C motif) ligand 1 (CXCL1), IL-1ß, nuclear factor-kappa B (NF-κB), inhibitor of NF-κB alpha (IκB-α), and NKCC1.

### Microvascular Permeability

The measurement of Kf in isolated lungs was conducted as previously described [11]. An index of Kf was determined from the lung weight change induced by elevated pulmonary venous pressure (PVP). The PVP was rapidly elevated by 10 cm H_2_O for 7 min. The slow, steady phase of weight gain as a function of time (ΔW/ΔT) was plotted on a semi-logarithmic paper and then extrapolated to zero time to obtain the initial rate of trans-capillary filtration. From this plot, Kf was defined as the *y*-intercept (gm/min) divided by PVP (10 cm H_2_O) and lung weight and expressed in whole units of grams per minute per centimeter of H_2_O multiplied by 100 g [28].

### Alveolar Fluid Clearance Measurement

AFC was measured using an *in situ* lung model as previously described [13]. In brief, the euthanized rats were maintained at 37–38 °C using a heating pad, and the lungs were inflated with 100% O_2_ at 7 cmH_2_O continuous positive airway pressure throughout the experiment. Subsequently, 12.5 mL/kg body weight of instillate containing fluorescein isothiocyanate (FITC)-conjugated albumin (Sigma-Aldrich, St. Louis, MO, USA) was delivered into the lungs over 1 min. An alveolar fluid sample (100 μL) was aspirated 1 min after instillation and 15 min later. The aspirates were centrifuged at 3000×*g* for 10 min, and the fluorescence activity in the supernatant was measured in duplicate. AFC was computed from the increase in alveolar fluid albumin concentration using the equation:

$$ \mathrm{AFC}=\left( Cf- Ci\right)/ Cf\times 100 $$

where *Ci* and *Cf* represent the initial and final concentrations of FITC-albumin in the aspirate at 1 and 15 min, as assessed by the fluorescence activity measurements.

### Pro-inflammatory Cytokines Levels in Perfusate

The expression levels of pro-inflammatory cytokines including TNF-α, CXCL1, and IL-1ß were determined by commercially available enzyme-linked immunosorbent assays (ELISA) (R&D Systems Inc., Minneapolis, MN).

### Pulmonary Edema

The lung W/D ratio was used as an indicator of pulmonary edema. After the experiment, part of the right middle lobe was weighed and then dried in an oven at 60 °C for 48 h. The wet and dry weights were then measured to calculate the lung W/D ratio.

### Lung Histopathology

Histopathologic examination was performed to verify the micro-anatomic features of AE-induced ALI and to assess the effects of surfactant. After the experiment, the lungs were removed and fixed with 10% formaldehyde at 20 cmH_2_O infused through the trachea. The tissues were immersed in 10% formaldehyde fixative for 24 h, embedded in paraffin wax, and cut into 4–6 μm-thick sections using a microtome. The sections were then stained with hematoxylin and eosin (H&E) to assess interstitial edema, inflammation, and degree of neutrophilic infiltration.

### Tissue Neutrophil Quantification

For tissue neutrophilic quantification, H&E-stained sections were used to count the number of neutrophils per high power field (× 400) [23]. For each slide, neutrophils were counted in 10 non-overlapping high power fields [9].

### Immuno-blotting

Cytoplasmic and nuclear proteins were extracted from frozen lung tissues using the Nuclear/Cytosol Extraction kit (BioVision, Inc., Mountain View, CA). Protein concentrations were determined by BCA protein assays and equal amounts of lung homogenates (30 μg/lane) were fractionated on 10–12% sodium dodecyl sulfate-polyacryl-amide gel electrophoresis (SDS-PAGE) gels and transferred to Hybond polyvinylidene fluoride membranes. The membranes were blocked by incubation in phosphate-buffered saline containing 0.1% Tween 20 and 5% non-fat milk for 1 h at room temperature.

The blots were incubated with antibodies against phosphorylated NF-κB p65, IκB-α and NKCC1 (Cell Signaling Technology, Danvers, MA) overnight at 4 °C. The blots were then washed three times for 10 min using phosphate-buffered saline containing 0.1% Tween 20. The blots were incubated with horseradish peroxidase linked anti-rabbit immunoglobulin G (1:40,000) or anti-goat immunoglobulin G (1:50,000) for 1 h at room temperature, and then washed three times in phosphate-buffered saline containing 0.1% Tween 20 for 10 min. The bands were visualized using enhanced chemiluminescence reagents and by exposing the blot to a radiography film. The blots were then stripped and incubated with an anti-TATA antibody (for nuclear protein, diluted 1:1000; Abcam, Cambridge, MA) or anti-β-actin antibody (for cytoplasmic protein, diluted 1:10,000; Sigma, St. Louis, MO) to ensure equal loading.

### Data Analysis

All statistical analyses were performed using the SPSS software 18.0 (SPSS Inc., Chicago, IL). Differences between groups were evaluated using Kruskal–Wallis followed by *post hoc* comparisons with Games–Howell tests (intergroup comparison). Statistical significance was set at *p* < 0.05.

## RESULTS

### Surfactant Decreased Air Embolism-Induced Pulmonary Edema (Fig. 1)

**Fig. 1:**
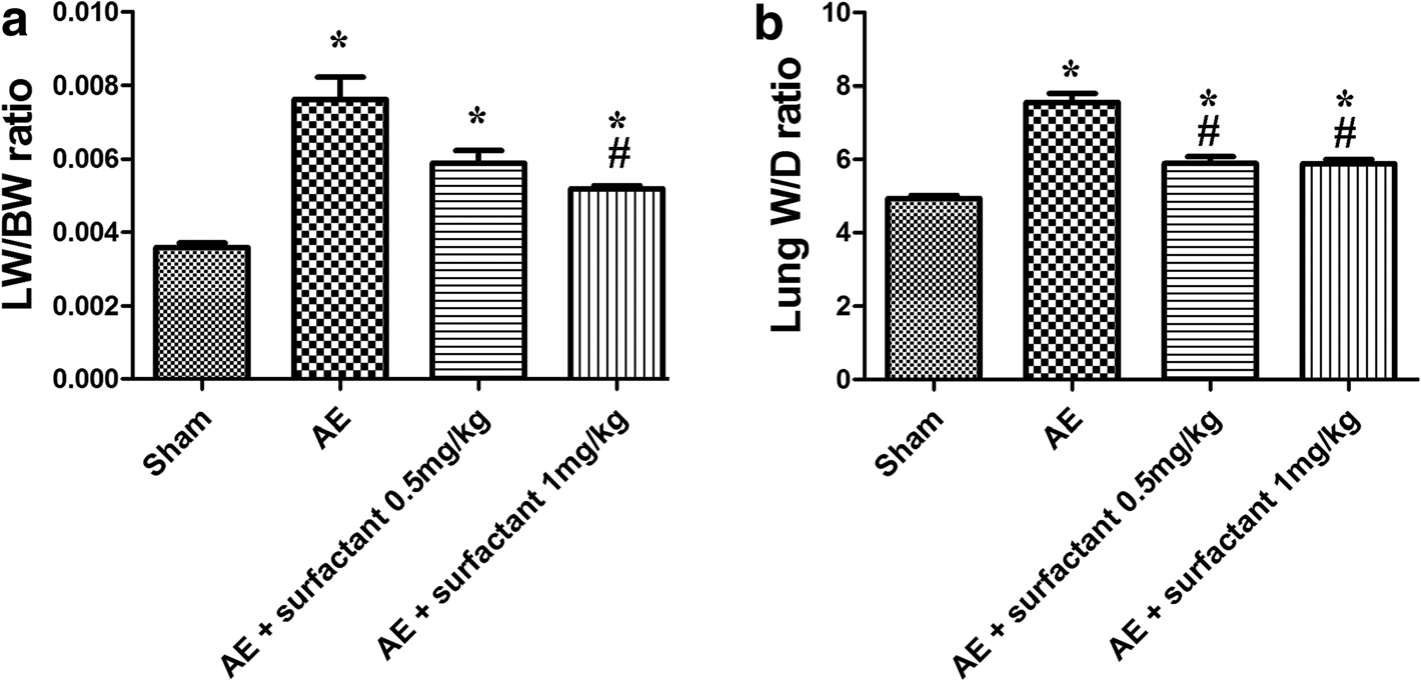
Surfactant decreased air embolism-induced pulmonary edema. **a** Lung weight/body weight (LW/BW) and wet/dry weight ratio (W/D) were significantly increased in the air embolism (AE) group compared to the sham group (*p* < 0.05). Surfactant 1 mg/kg significantly decreased LW/BW and W/D compared to the AE group (*p* < 0.05). There was a significant difference between the *Sham (*p* < 0.05) and ^#^AE (*p* < 0.05) groups. Abbreviations: AE, air embolism; LW/BW, lung weight/body weight ratio; W/D: wet/dry weight ratio.

Both LW/BW ratio (Fig. 1a) and lung W/D ratio (Fig. 1b) were significantly increased in the AE group as compared to the sham group (*p* < 0.05). Surfactant 1 mg/kg significantly decreased AE-induced pulmonary edema as compared to the AE group (*p* < 0.05).

### Surfactant Decreased Air Embolism-Induced Microvascular Hyper-Permeability (Fig. 2)

**Fig. 2:**
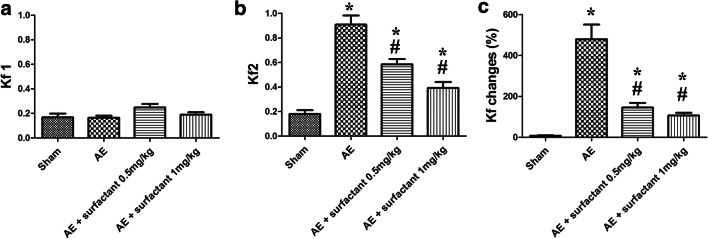
Surfactant decreased air embolism-induced pulmonary microvascular hyper-permeability. **a** Pulmonary microvascular permeability at baseline (Kf1) was similar among these groups (*p* > 0.05). **b** Pulmonary microvascular permeability after air embolism (Kf2) was significantly increased in the air embolism (AE) group. Surfactant 1 mg/kg significantly decreased post-AE microvascular permeability, compared to the AE group (*p* < 0.05). **c** Changes in pulmonary microvascular permeability (Kf) were prominent in the AE group and attenuated in rats with surfactant 1 mg/kg compared to the AE group (*p* < 0.05). There was a significant difference between the *Sham (*p* < 0.05) and ^#^AE (*p* < 0.05) groups. Abbreviations: AE, air embolism; Kf1, pulmonary microvascular permeability at baseline; Kf2, pulmonary microvascular permeability after AE.

The Kf1 at baseline was similar among these groups (*p* > 0.05) (Fig. 2a). The Kf2 was significantly increased in the AE group compared to the sham group (*p* < 0.05). Surfactant 1 mg/kg significantly decreased post-AE microvascular permeability, compared to the AE group (*p* < 0.05). The changes of Kf between baseline and post-AE were more prominent in the AE group compared to the sham group (*p* < 0.05), and were attenuated in the rats treated with surfactant 1 mg/kg (*p* < 0.05).

### Surfactant Restored Alveolar Fluid Clearance (Fig. 3)

AFC is an indicator of water re-absorption from the alveolar space into the interstitium. AFC was significantly lower in the AE group compared to the sham group (*p* < 0.05), and surfactant 1 mg/kg significantly restored AFC (*p* < 0.05, compared with the AE group).

**Fig. 3:**
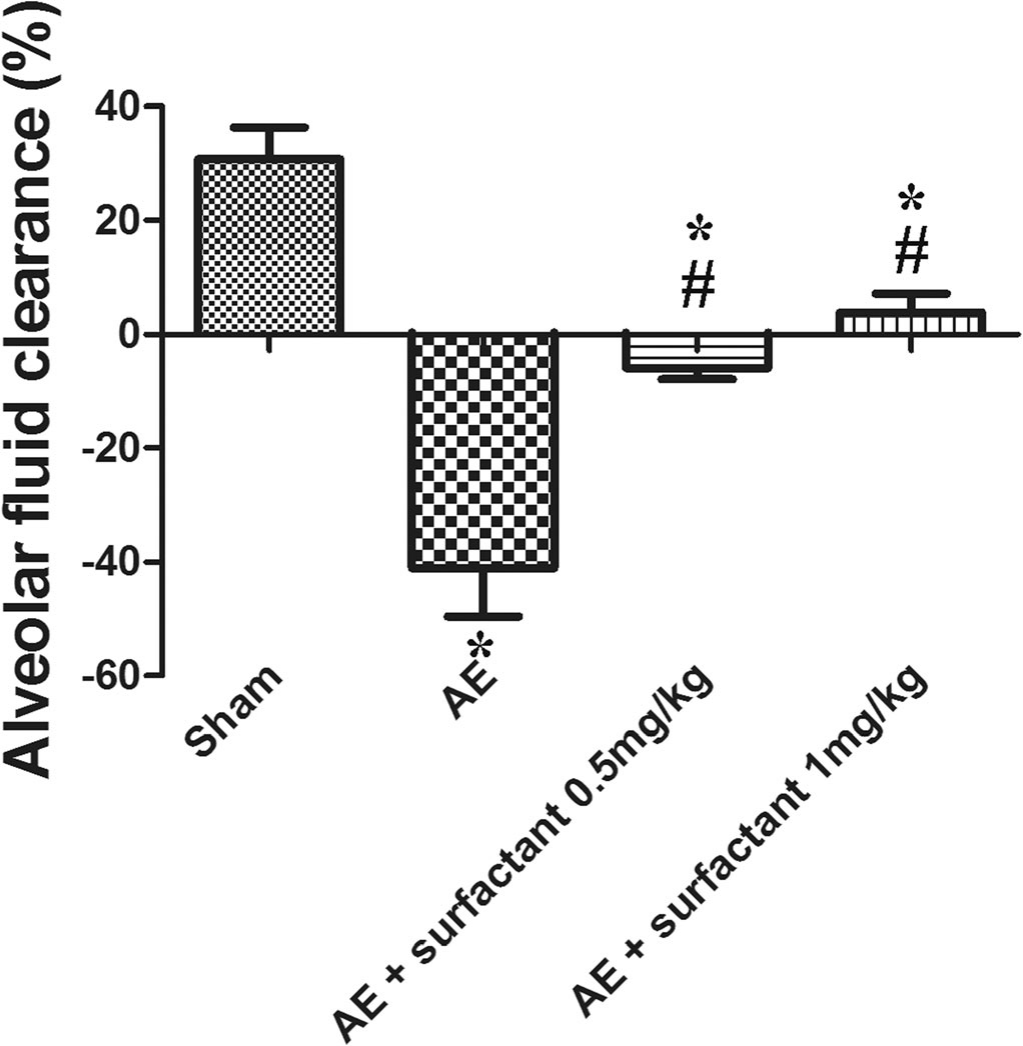
Surfactant restored alveolar fluid clearance. Alveolar fluid clearance (AFC) decreased markedly in the air embolism (AE) group, compared to the sham group (*p* < 0.05). Surfactant 1 mg/kg significantly restored AFC, compared to the AE group (*p* < 0.05). There was a significant difference between the *Sham (*p* < 0.05) and ^#^AE (*p* < 0.05) groups.

### Surfactant Attenuated Lung Injury (Fig. 4)

**Fig. 4:**
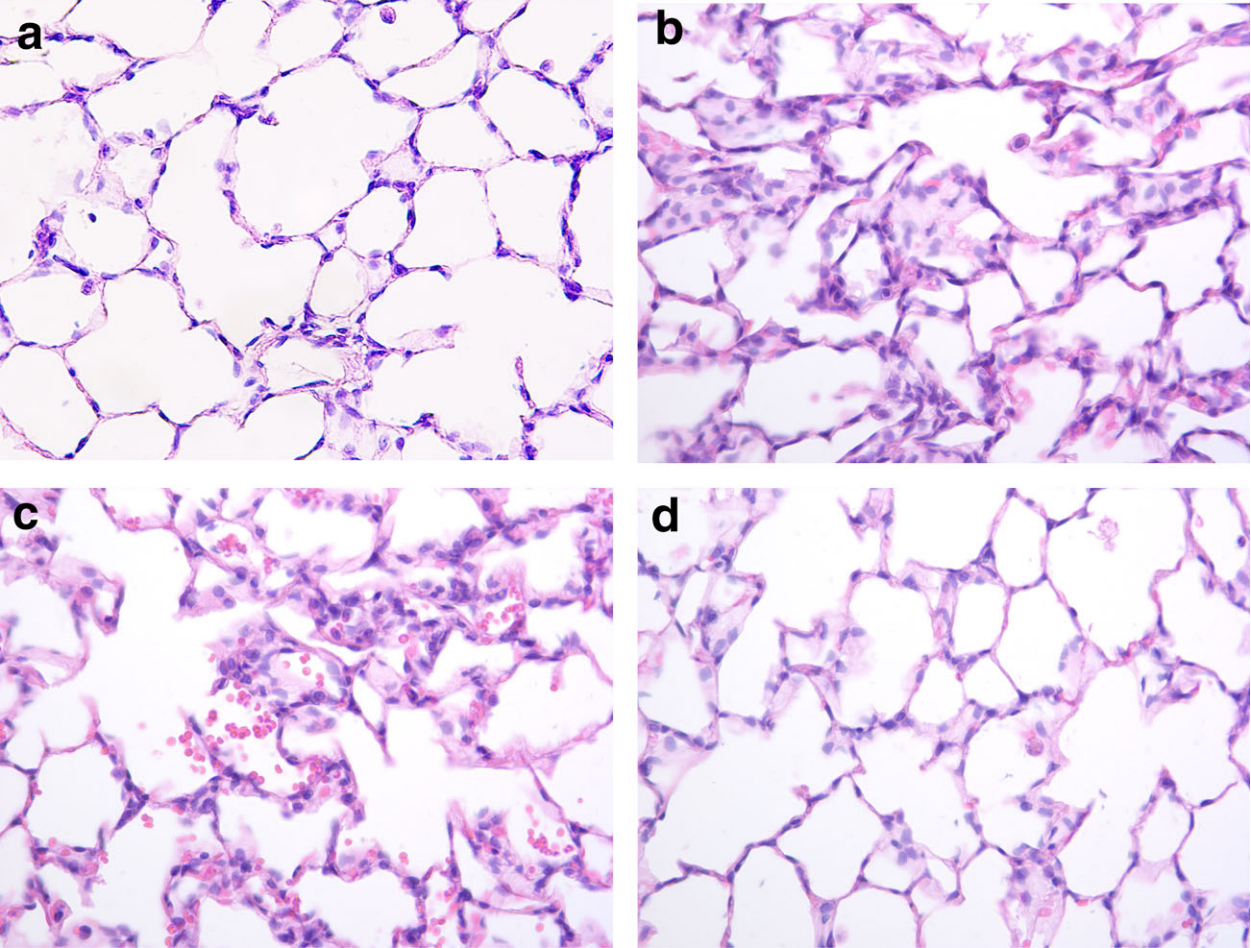
Surfactant attenuated lung injury. **a** The rats in the sham group had normal histology, **b** while the air embolism (AE) group showed prominent neutrophilic sequestration and inter-alveolar septum thickening. **c** Lung injury was less severe in rat with surfactant 0.5 mg/kg and **d** was markedly attenuated by surfactant 1 mg/kg.

The rats of the sham group had normal histology (Figs. 4a), while the AE group showed prominent neutrophilic sequestration and inter-alveolar septum thickening (Fig. 4b). Lung injury was less severe in rats receiving surfactant 0.5 mg/kg (Fig. 4c) and was markedly attenuated by surfactant 1 mg/kg (Fig. 4d).

### Surfactant Attenuated Lung Injury and Neutrophilic Sequestration (Fig. 5)

Quantified neutrophilic counts showed significantly higher numbers of neutrophils in the AE group compared to the sham group (*p* < 0.05); the numbers were significantly reduced by the administration of surfactant 1 mg/kg (*p* < 0.05).

**Fig. 5:**
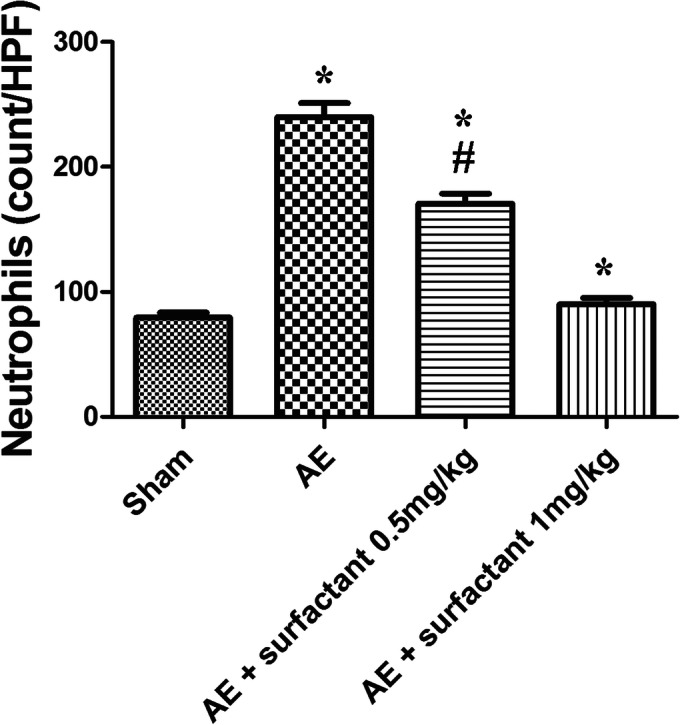
Surfactant attenuated neutrophilic sequestration. Neutrophilic count in lung tissues was markedly increased in the air embolism (AE) group, compared to the sham group (*p* < 0.05), and significantly reduced by surfactant 1 mg/kg, compared to the AE group (*p* < 0.05). There was a significant difference between the *Sham (*p* < 0.05) and ^#^AE (*p* < 0.05) groups.

### Surfactant Decreased Expressions of Pro-inflammatory Cytokines (Fig. 6)

**Fig. 6:**
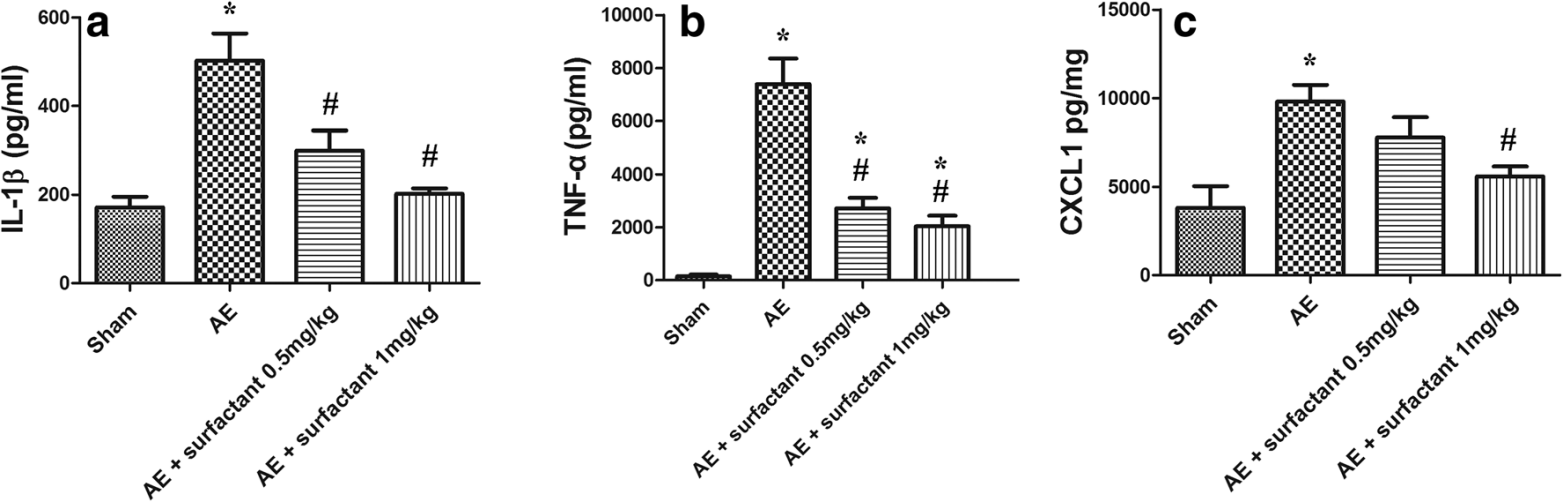
Surfactant decreased expressions of pro-inflammatory cytokines. Expression levels of pro-inflammatory cytokines, **a** IL-1ß, **b** TNF-α, and **c** CXCL-1 were significantly increased in the air embolism (AE) group, compared to the sham group (*p* < 0.05). Surfactant 1 mg/kg decreased expressions of these cytokines after AE (*p* < 0.05). There was a significant difference between the *Sham (*p* < 0.05) and ^#^AE (*p* < 0.05) groups. Abbreviations: AE, air embolism; IL-1ß: Interleukin-1ß; TNF-α: tumor necrosis factor-α; CXCL-1: chemokine (C-X-C motif) ligand 1.

Expression levels of pro-inflammatory cytokines, IL-1ß (6a), TNF-α (Fig. 6b), and CXCL-1 (6c) were significantly increased in the AE group compared to the sham group (*p* < 0.05). Surfactant 1 mg/kg decreased the expressions of these cytokines after AE (*p* < 0.05).

### Surfactant Decreased Air Embolism-Induced Expression of NF-κB Activation and Nuclear Translocation (Fig. 7)

**Fig. 7:**
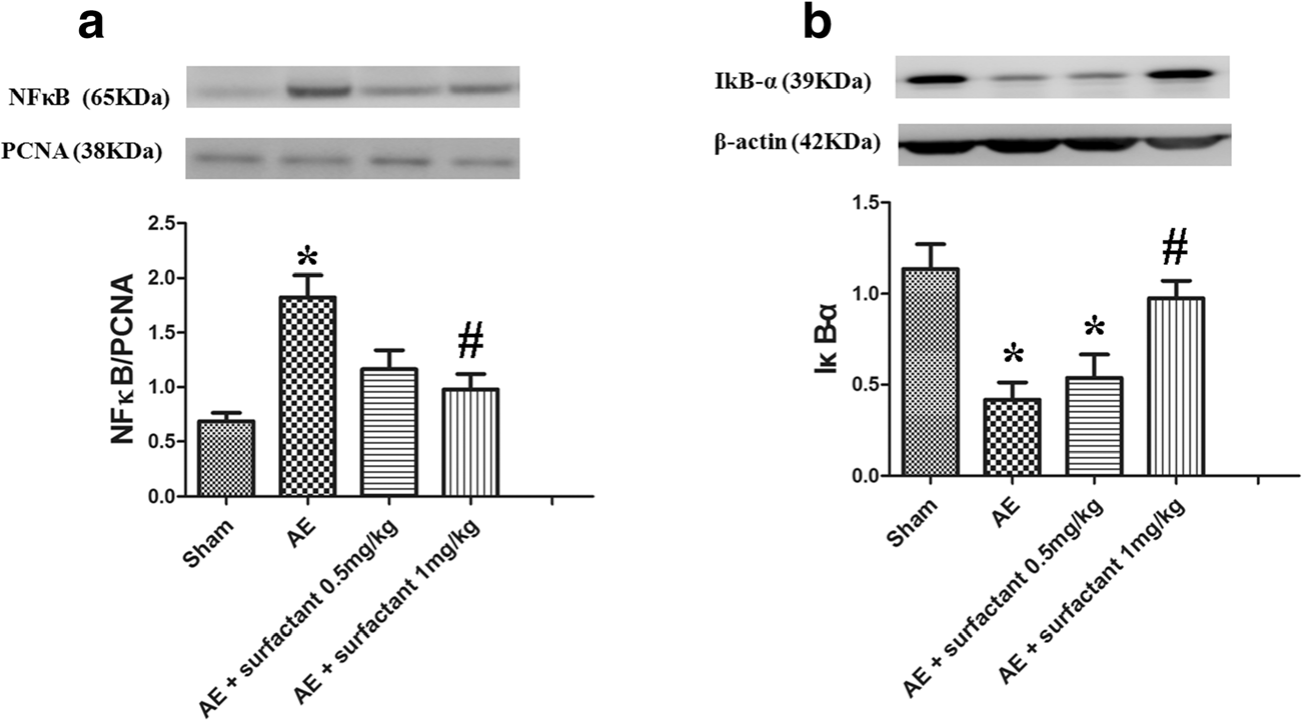
Surfactant decreased air embolism-induced expression of NF-κB activation. In the air embolism (AE) group, the cytoplasmic level of phosphorylated NF-κB p65 **a** was increased, whereas IκB-α **b** was significantly suppressed compared to the sham group (*p* < 0.05). Surfactant 1 mg/kg reduced phosphorylated NF-κB p65 and restored IκB-α compared to the AE group (*p* < 0.05). There was a significant difference from the *Sham (*p* < 0.05) and ^#^AE (*p* < 0.05) groups. Abbreviations: AE, air embolism; NF-κB, nuclear factor-kappa B; IκB-α, inhibitor of NF-κB alpha.

In the AE group, the cytoplasmic level of phosphorylated NF-κB p65 (Fig. 7a) was increased, whereas that of IκB-α (Fig. 7b) was significantly suppressed as compared to the sham group (*p* < 0.05). Surfactant 1 mg/kg reduced phosphorylated NF-κB p65 level and restored IκB-α level as compared to the AE group (*p* < 0.05).

### Surfactant Decreased Air Embolism-Induced Expression of NKCC1 (Fig. 8)

The expression of NKCC1 was significantly increased in the AE group as compared to the sham group (*p* < 0.05). Surfactant 1 mg/kg significantly decreased the expression of NKCC1, compared to the AE group (*p* < 0.05).

**Fig. 8:**
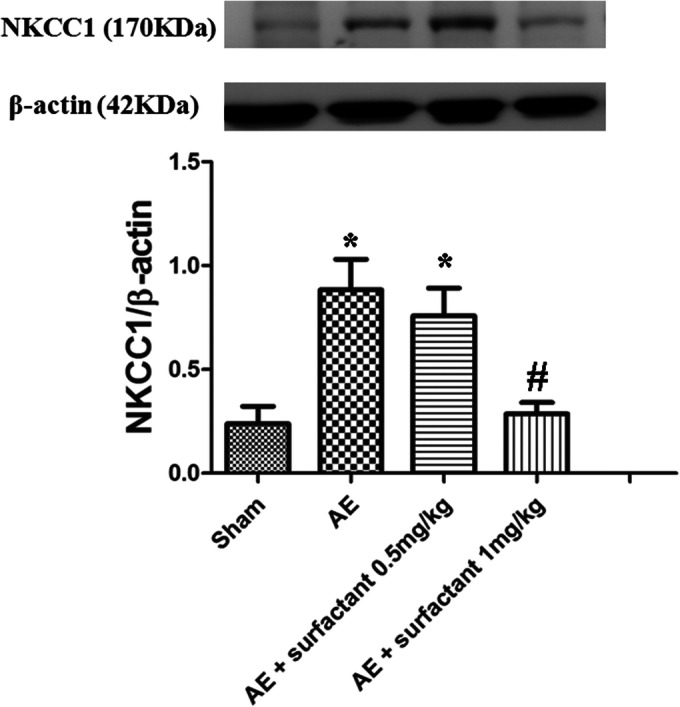
Surfactant decreased air embolism-induced expression of NKCC1. The expression of NKCC1 was significantly increased in the air embolism (AE) group compared to the sham group (*p* < 0.05). Surfactant 1 mg/kg significantly decreased the expressions of NKCC1 compared to the AE group (*p* < 0.05). There was a significant difference between the *Sham (*p* < 0.05) and ^#^AE (*p* < 0.05) groups. Abbreviations: AE, air embolism; Na-K-Cl cotransporter 1.

## DISCUSSION

In the current study, AE-induced ALI showed pulmonary edema, pulmonary microvascular hyper-permeability, and lung inflammation with neutrophilic sequestration. AE-induced lung injury also presented with increased expressions of pro-inflammatory cytokines, activation of NF-κB, and NKCC1. Surfactant administration attenuated AE-induced lung hyper-permeability, pulmonary edema, and lung inflammation with concurrent decreased expression of cytokines, NF-κB, and NKCC1. These results suggest the therapeutic effects of surfactant in AE-induced ALI.

It is interesting to discuss the mechanisms of the protective effects of surfactant in AE-induced ALI. Surfactant is important in the pathogenesis of ALI. Surfactant dysfunction contributes to the progressive loss of aerated volume, lung compliance, and worsening ventilation [6]. Surfactant aids in the reduction of alveolar surface tension and maintains alveolar opening and prevents alveolar collapse [20]. Hence, the surfactant can improve lung ventilation and attenuate the progression of lung injury [6]. We reveal that surfactant also protects against AE-induced lung injury.

Surfactant has immunomodulatory features with regulating pulmonary innate and acquired immunity and inflammatory processes [3]. Surfactant is known to decrease the release of pro-inflammatory cytokines and chemokines and further decrease lung inflammatory cells such as neutrophils, monocytes, and macrophages [3]. Therefore, therapeutic efficacy of the surfactant is related not only to its biophysical characteristics but also due to its anti-inflammatory features [3]. In this study, we confirm that the surfactant decreases pro-inflammatory cytokines and neutrophils sequestration in AE-induced ALI.

NF-κB is a key transcription factor in cytokine gene expression and is important in ALI [11, 27]. Our results show activation of NF-κB pathway in AE-induced ALI. When cells are activated by inflammatory stimuli such as AE, IκB is phosphorylated by IKK complex proteins [27]. Subsequently, NF-κB binds to κB enhancer elements of target genes to induce the transcription of inflammatory genes and increase expression of inflammatory cytokines [27]. The current study reveals the concurrent activation of NF-κB and pro-inflammatory cytokines after AE. After the administration of the surfactant, activation of NF-κB and pro-inflammatory cytokines was decreased.

Neutrophils play an essential role in the onset of ALI [5]. Activation of NF-κB pathway leads to increased expression of cytokines [27]. Pro-inflammatory cytokines have a chemotactic effect and lead to migration of neutrophils into the air spaces [27]. Migratory neutrophils further degranulate and release toxic intracellular products, free radicals, and cytotoxic enzymes that lead to lung injury [5, 8]. Surfactant decreases activation of NF-κB and pro-inflammatory cytokines, thereby decreases neutrophil sequestration in lungs and attenuating the severity of lung injury.

The regulation of surfactant in alveolar ion channels and fluid clearance is largely unknown. AFC was decreased in AE-induced lung injury and surfactant restored AFC after lung injury. Restoration of AFC represents a lung-protection mechanism *via* which excessive fluid is removed from the alveoli to restore ventilation for gas exchange [13]. Surfactant aids in the reduction of alveolar surface tension and prevents lung interstitial proteins and fluids leaking into the alveolar cavity [20]. Therefore, it is rational that surfactant can restore AFC in AE-induced lung injury.

NKCC1 is important in the regulation of lung water and inflammation. In previous studies, expression of NKCC1 was found to be increased in hyperoxia and ischemia-reperfusion-induced lung injury [11, 13]. The animals with higher expression of NKCC1 showed more severe lung injury [10]. In this study, we also found increased expression of NKCC1 in AE-induced ALI. The activation of the IKK–NF-κB cascade causes osmotic stress and cell swelling, which can activate with-no-lysine kinase (WNK) kinases [21, 26]. Activation of WNK kinase has been shown to phosphorylate and activate NKCC1 [21].

NKCC1 influences both inflammatory responses and fluid regulation in the lungs [13, 16, 24]. The NKCC1 works to transport Cl^−^ and is involved in the regulation of epithelial cell volume [17]. Up-regulating NKCC1 leads to dysregulation of fluid transport, cellular swelling, and inflammation [16, 18]. NKCC1 regulates lung endothelial and epithelial barriers and modulates the inflammatory response to lung injury [13]. Therefore, increased expression of NKCC1 results in decreased alveolar liquid absorption, increased lung water, and inflammation. Conversely, inactivation of NKCC1 by surfactant leads to restored AFC, decreased lung water, and inflammation.

There are some limitations in our study. We suggest that the surfactant reduces the severity of AE-induced lung injury. This investigation provides a possible therapy in AE-induced lung injury in clinical settings. However, the present study has a few limitations. Firstly, the study was performed in animals using an isolated lung model. Further studies in human beings are warranted. Secondly, the present study is limited to the very early stage of AE-induced ALI. Further investigation is required to address the longer-term responses of surfactant in ALI.

### Clinical Implications

The lungs are vulnerable solid organs. Pulmonary AE is a rare but potentially fatal complication of invasive medical or surgical procedures [7]. Therefore, it is important to study the effective treatments in AE-induced ALI. In the current study, we suggested that the surfactant could attenuate AE-induced ALI. This investigation provides incentive to explore impetus to consider approaches that address the role of surfactant within this the clinical setting.

## CONCLUSIONS

AE-induced ALI presents with pulmonary edema, lung inflammation with neutrophilic sequestration, pulmonary microvascular hyper-permeability, and increased expressions of pro-inflammatory cytokines, NF-κB, and NKCC1. Surfactant attenuates pulmonary edema, lung inflammation, and down-regulates the expression of pro-inflammatory cytokines, NF-κB, and NKCC1. This study provides incentive to explore the effects of surfactant in the clinical setting. However, further investigation is still required to address the therapeutic effects of surfactant in ALI in humans.
